# Simultaneous Evaluation of Life Cycle Dynamics between a Host *Paramecium* and the Endosymbionts of *Paramecium bursaria* Using Capillary Flow Cytometry

**DOI:** 10.1038/srep31638

**Published:** 2016-08-17

**Authors:** Toshiyuki Takahashi

**Affiliations:** 1 Department of Chemical Science and Engineering, National Institute of Technology, Miyakonojo College, Miyazaki, Japan

## Abstract

Endosymbioses are driving forces underlying cell evolution. The endosymbiosis exhibited by *Paramecium bursaria* is an excellent model with which to study symbiosis. A single-cell microscopic analysis of *P. bursaria* reveals that endosymbiont numbers double when the host is in the division phase. Consequently, endosymbionts must arrange their cell cycle schedule if the culture-condition-dependent change delays the generation time of *P. bursaria*. However, it remains poorly understood whether endosymbionts keep pace with the culture-condition-dependent behaviors of *P. bursaria*, or not. Using microscopy and flow cytometry, this study investigated the life cycle behaviors occurring between endosymbionts and the host. To establish a connection between the host cell cycle and endosymbionts comprehensively, multivariate analysis was applied. The multivariate analysis revealed important information related to regulation between the host and endosymbionts. Results show that dividing endosymbionts underwent transition smoothly from the division phase to interphase, when the host was in the logarithmic phase. In contrast, endosymbiont division stagnated when the host was in the stationary phase. This paper explains that endosymbionts fine-tune their cell cycle pace with their host and that a synchronous life cycle between the endosymbionts and the host is guaranteed in the symbiosis of *P. bursaria*.

Studying symbiotic associations sheds light on cell-to-cell interaction, parasitic diseases, and evolution of eukaryotic cells. Endosymbiosis that performs functions derived from symbionts to the host organisms is a driving force underlying cell evolution. Photosynthesis takes place in some bacteria (cyanobacteria), chlorophyll-containing algae, and plants. Primary green algae, red algae, and land plants are particularly derived from evolutionary results in a primary endosymbiosis between a cyanobacterium and an ancestral eukaryotic cell^1^,^2^,^3^,^4^. The genes for a functional plastid flowed as endosymbiotic gene transfer (EGT) from cyanobacterial endosymbiont to host, thereby depriving the endosymbiont of autonomous control^5^,^6^,^7^,^8^,^9^,^10^,^11^. Following primary endosymbiosis, secondary and tertiary endosymbioses produced some algae including Euglenophyta and Chlorarachniophyta with the plastids of green algae as photobionts, and other algae including Heterokontophyta, Haptophyta, Cryptophyta, and Dinophyta with those of red algae^2^,^3^,^8^. To achieve stable endosymbiosis, some changes must occur in both the eukaryotic host and the eukaryotic symbionts^8^: i. establishment of a specific partner alga; ii. lateral gene transfer (LGT) from eukaryotic symbionts to the host nucleus^11^; iii. development of protein-transport machinery to carry proteins from host cytoplasm to the symbiont; and iv. synchronization of division cycles.

Several endosymbioses, outside of those above completed secondary and tertiary endosymbioses, are presently progressing. These symbioses are also accomplished by multistep processes as well as the primary, secondary, and tertiary endosymbioses: 1. Infection of symbionts into a host organism; 2. Recognition of symbionts by the host; and 3. Stable establishment of symbiosis. These representative symbioses are observed between freshwater hosts and algal symbionts in *Paramecium bursaria*^12^,^13^,^14^,^15^,^16^ and *Hydra viridissima*^12^,^17^,^18^ (i.e. *Hydra viridis*), between seawater hosts and algal symbionts in coral^12^, anemone, sponge^19^,^20^,^21^,^22^,^23^ and the nudibranch *Melibe engeli*^24^. Other symbioses are also observed between hosts and transient plastids from algal prey in a marine ciliate *Mesodinium rubrum*^25^,^26^,^27^,^28^ (i.e. *Myrionecta rubra*), a sacoglossan sea slug such as *Elysia chlorotica*, and *Elysia timida*^4^,^29^,^30^,^31^.

One ciliate, *P. bursaria*, has several hundred *Chlorella*-like endosymbionts in its cytoplasm^32^,^33^ (Fig. 1A). Treatment of *P. bursaria* with several chemicals such as herbicide paraquat^34^ and acrylamide^35^,^36^ or by incubation in continuous dark condition^37^ can disorder the symbiotic association in *P. bursaria*. Such treatments can eventually produce algae-free paramecia (Fig. 1B). Apparently, their endosymbiotic algae are not absolutely necessary for *P. bursaria* host cells because the algae-free paramecia can proliferate in media containing food bacteria independently of the endosymbiotic algae. The sexual reproduction ability (conjugation ability) of algae-free *Paramecium* is apparently unaffected by the loss of endosymbiotic algae (Fig. 1C,D). When the algae-free *Paramecium* is mixed with exosymbiotic algae isolated from *P. bursaria*, the algae-free *Paramecium* takes algae and reconstructs the symbiotic relation^32^,^37^,^38^. The expression “exosymbiotic alga” in this report denotes a cloned alga that is isolated from *P. bursaria*. Based on these features, the endosymbiosis in *P. bursaria* is introduced as an excellent model with which one can study endosymbiosis experimentally. Although many symbiotic studies using *P. bursaria* have taken particular note of infection processes, including the host’s recognition of symbionts^32^,^37^, less attention has been devoted to how the symbiotic relation between host and their endosymbionts is maintained stably. However, the coordination of host-symbiont division as well as the influence of external factors on *P. bursaria* are worth clarifying, as earlier studies have done for eukaryotic organelles of endosymbiotic evolutionary origin^9^,^10^.

Over the last several decades, flow cytometry (FCM), a powerful and valuable tool for studies of cell biology and microbiology, has become widely used. Based on hydrodynamic focusing with a sheath fluid, this technique can assess cell optical information. Moreover, FCM has functions that include several procedures such as cell counting, detection of biomarkers, and cell sorting. In fact, FCM can save more time and provide higher sensitivity to optical properties than microscopic observation can. These features of FCM have provided an important boost to the apparatus as a driving force for important breakthroughs in science, protein engineering, and healthcare. Commercial flow cytometers with standard flow cell tips having diameters of 50–72 μm^39^ are generally optimized for standard suspended cells with 1–30 μm diameter^40^. Using flow cytometry is challenging for analysis and sorting of larger cells, even plant cells, which are large (*ca*. 20–100 μm), thereby limiting the use of a typical nozzle in commercial flow cytometry^41^. For a smooth and clog-free run, the flow nozzle should be at least four times the objective particle size^41^. Therefore, the FCM technique has been used only rarely to investigate complex systems between eukaryote cells and small eukaryotic symbionts such as *P. bursaria*, which is a large cell for examination using conventional FCM technique. Instead, exosymbiotic algae isolated from *P. bursaria*, but not host cells, have been analyzed mainly using FCM^42^ because it has been assumed that host ciliates are damaged and broken by the passage pressure of hydrodynamic focusing during FCM measurements using standard flow cell tips. Actually, *P. bursaria*, having cell width and length of *ca.* 50 μm and 120–150 μm, is larger than the suspended cells applicable to standard FCM. Some reports of the literature describe the flow cytometric analysis of *P. bursaria* using a special size instrument nozzle of 200 μm diameter^43^. However, many data obtained only from their algae are insufficient to elucidate the mechanisms of endosymbiosis because algae have already been disengaged from control by the host. A recent study has demonstrated that capillary FCM, not hydrodynamic focusing, can detect intact *P. bursaria*^44^. That fact implies that this system might be useful as a powerful tool for studies of symbioses such as that shown by *P. bursaria*.

This report presents a method of simultaneous detection of life cycle dynamics between hosts and endosymbionts of *P. bursaria* using capillary FCM with no special size nozzle. This study evaluated the relation between the host and endosymbiont life cycle using multivariate principal component analysis (PCA). Results show that the host cell cycle accommodated the cell cycle pace of endosymbiotic algae.

## Results

### Detection of population dynamics of *P. bursaria* host cells using capillary FCM

**Figure 2:**
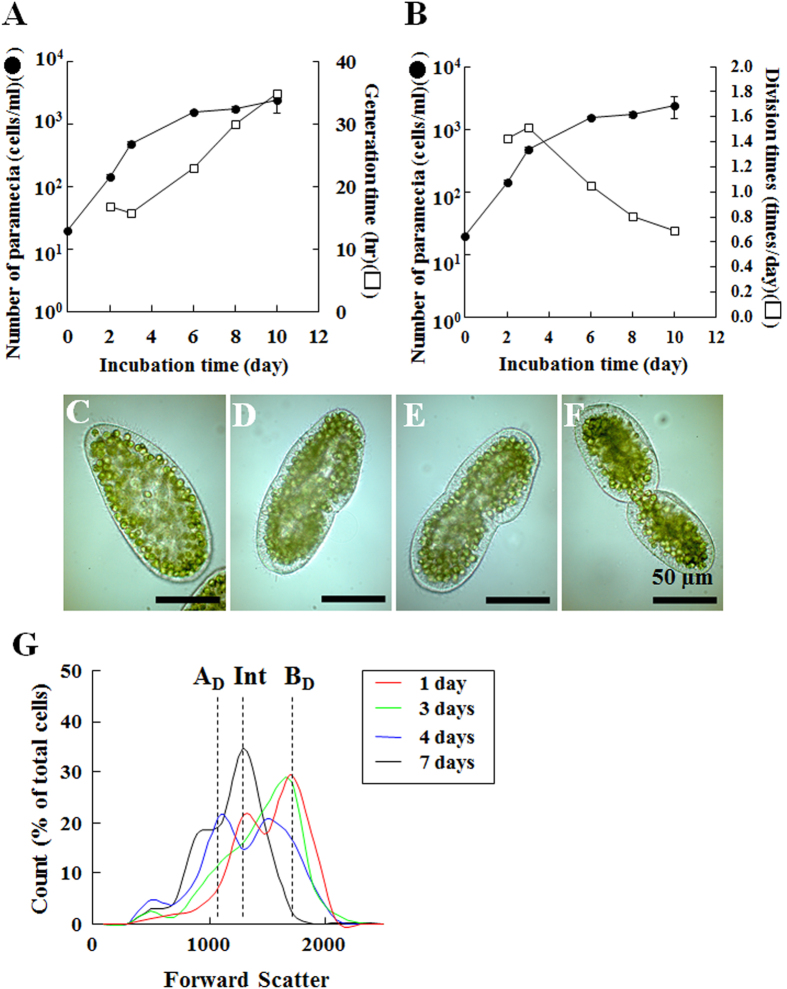
Growth dynamics of *P. bursaria* host cells. (**A**,**B**) Time-dependent population dynamics of *P. bursaria* were tracked over time. Then, the generation time and division times were estimated. (**C–F**) Microscopic images of *P. bursaria* at interphase and division phase. (**G**) Data obtained with *P. bursaria* possessing endosymbiotic algae were reconstructed using external software for FSS vs. count (% of total signals for intact *P. bursaria* cells). Dotted lines in the panel G represent each detectable and distinguishable peak size of *P. bursaria*. Here, the population of small cells of *P. bursaria* immediately after cell division (dotted line, A_D_), that of cells at cytokinesis as in Fig. 2F (dotted line, B_D_), and that of the intermediate cell sizes at the interphase or nuclear division phase (dotted line, Int) were detected using FCM.

Before evaluating the coordination between the host and endosymbiont division, the growth of *P. bursaria* host cells was tracked (black circle in Fig. 2A). In addition to the total number of host cells, the generation time (white squares in Fig. 2A) was calculated. Here, the generation time of *P. bursaria* signifies the time-to-next division after the prior division. When the number of paramecia increased logarithmically and reached the stationary phase, the generation time was extended gradually from *ca.* 16 hr to more than 24 hr. This delayed generation time was fundamentally equal to the reduction of the division time of *P. bursaria* (white squares in Fig. 2B).

Cell sizes of *P. bursaria* change slightly, as shown in Fig. 2C–F, through the *Paramecium* cell cycle: *P. bursaria* is depicted at the interphase (Fig. 2C), paramecia are presented in Fig. 2D,E at the nuclear division phase; and *Paramecium* is portrayed at cytokinesis (Fig. 2F). The *P. bursaria* presented in Fig. 2F is divided into two daughter cells a few minutes later. This study was conducted to detect cell cycle-dependent sizes using capillary FCM. To extract signals derived from host paramecia using FCM, the gating method described in a previous study^44^ was adopted. Results revealed that three distinguishable populations were detected according to cell size based on forward scatter signals (FSS) (Fig. 2G): The population of small cells of *P. bursaria* immediately after cell division (dotted line, designated as A_D_), that of cells at cytokinesis as in Fig. 2F (dotted line, B_D_), and that of the intermediate cell sizes at the interphase or nuclear division phase (dotted line, Int). FCM data from paramecia at the early stationary phase converged on the intermediate sizes, whereas those at the log phase (1-day to 4-day of incubation) diverged from the population for small cells after cell division to that at cytokinesis. Consequently, FCM was able to detect the cell-cycle-dependent size change of *P. bursaria*.

### Detection of population dynamics of endosymbionts in *P. bursaria* using capillary FCM

Gradual reduction of the proliferation activity of *P. bursaria* (Fig. 2A,B) is generally related both to depletion of nutritional resources and to deterioration of culture conditions because of cell waste. Microscopic comparison of the number of endosymbionts in individual *P. bursaria* at the interphase with that at the division phase in earlier studies^15^,^45^ has shown that *P. bursaria* at the division phase had almost double the number of endosymbionts as those present at the interphase. However, it remains unclear whether the endosymbionts have a growth rate that matches the culture-condition-dependent reduction of the host proliferation activity.

**Figure 3:**
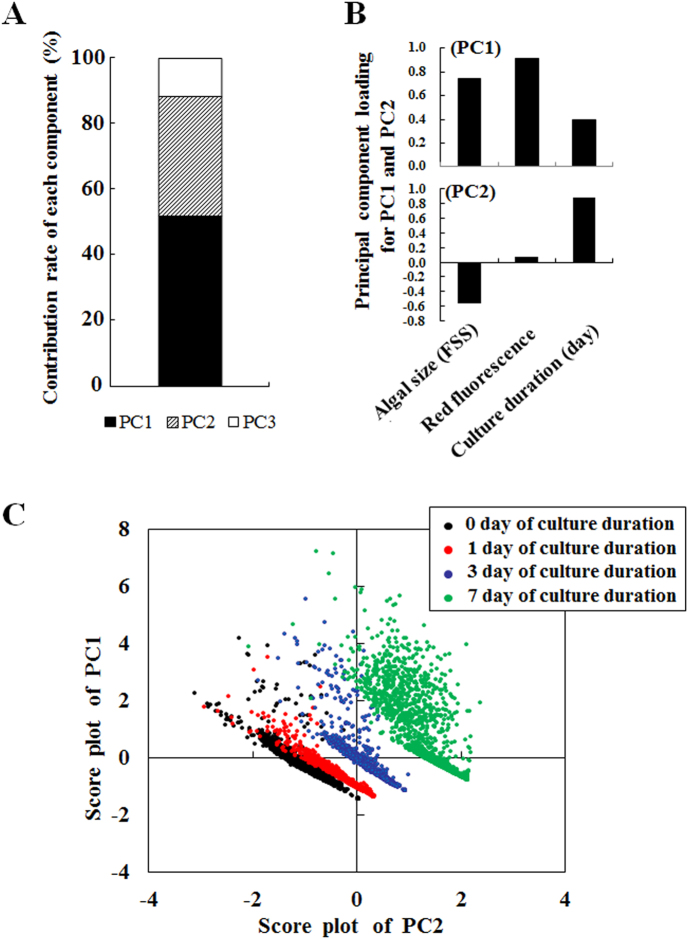
Cell cycle dynamics of endosymbionts in *P. bursaria* by PCA method. (**A**) The PCA reduces multiple-dimensional information to arbitrary one-dimensional information and produces new components such as PC1-PC3. Each contribution rate of each component was expressed as a stacked bar graph. (**B**) Factor loading plots of each parameter for PC1 and PC2, respectively. (**C**) Score plots of PC1 vs. PC2 were produced using data from different culture durations.

To confirm the optical properties of endosymbionts throughout the population dynamics of *P. bursaria*, FCM was used for this study. To extract signals for endosymbionts of *P. bursaria* from FCM data including host signals, gating strategies for endosymbionts described in the previous study^44^ were used. Whereas the size and chlorophyll contents of host cells were evaluated from the R1 population for intact *P. bursaria* host cells, those for endosymbiotic algae were evaluated simultaneously from the R2 population for endosymbiotic algae (see *Methods*). First, this study evaluated correlation between the host and algal life cycle using PCA of multivariate analysis. The dataset from FCM data was subjected to PCA method, which reduces multiple-dimensional information to arbitrary one-dimensional information. The primary (PC1), secondary (PC2), and tertiary (PC3) components respectively presented 51.7%, 36.5%, and 11.8% of information in data from the contribution rate (Fig. 3A). Figure 3B presents the principal component loading of PC1 and PC2. Each loading showed that all parameters, including the algal size (FSS), red fluorescence intensity, and culture time of *P. bursaria* (Day), were positively correlated with PC1 (Fig. 3B). Particularly, correlation factors for both the algal size and the red fluorescence intensity were more strongly positive with PC1 than the culture duration of *P. bursaria* was. By contrast, the algal size and the culture duration respectively showed inverse and positive correlation with PC2. Figure 3C presents the culture condition-dependent variation of endosymbionts as expressed by the score plot of PC1 versus PC2. The characteristics of both algal size and red fluorescence intensity were mainly reflected as the variation of endosymbionts on the positive PC1-axis (Fig. 3B), whereas only the culture duration of *P. bursaria* mainly affected the variation of endosymbionts on the positive PC2-axis (Fig. 3B). This result reflects that the variation of endosymbionts changed the culture duration of *P. bursaria* dependently (Fig. 3C). Results show that both the cell size and chlorophyll fluorescence intensity of endosymbionts can be an indicator to assess the variation.

**Figure 4:**
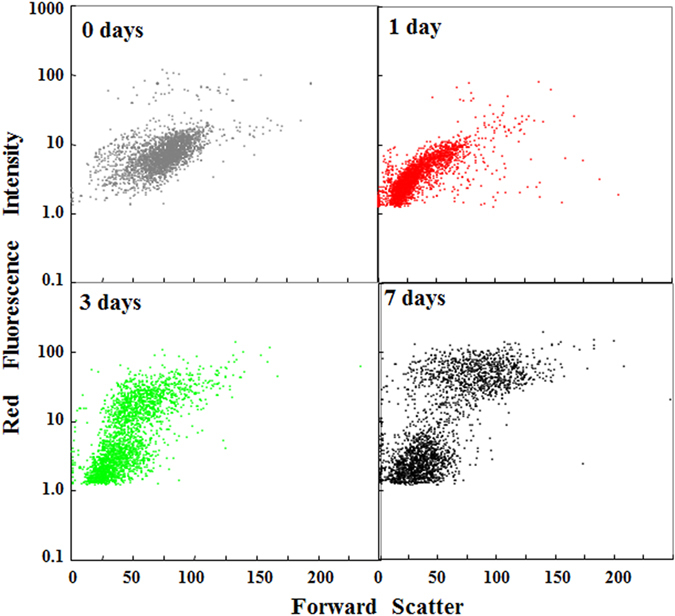
Population distribution of endosymbionts in *P. bursaria* using FCM. The obtained data were reconstructed to produce a dot plot for FSS vs. red fluorescence intensity (% of total signals for endosymbiotic algae).

**Figure 5:**
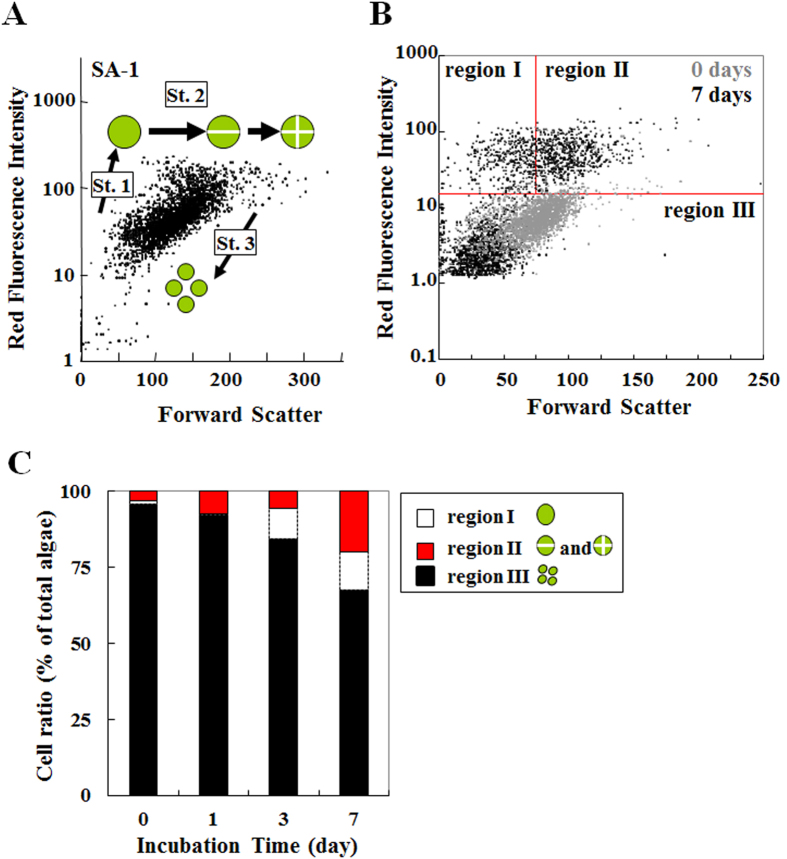
Analysis of population distribution dynamics of endosymbiotic algae in *P.* *bursaria*. (**A**) Population distribution of exosymbiotic algae (SA-1) as a reference^46^. St. 1, “growth” stage; St. 2, “ripening” stage; and St. 3, “division and autospore liberation” stages. (**B**) Comparison among distribution patterns of endosymbiotic algae was made culture term by culture term. Based on algal optical properties that have been related to the algal cell cycle, the distribution patterns of endosymbionts were categorized into three populations (regions I-III). (**C**) Time-dependent changes of algal distribution patterns were estimated as the stacked bar graph at each incubation time of *P. bursaria*.

The PCA analysis results (Fig. 3) prompted us to produce plots of FSS for endosymbionts versus the red fluorescence intensity for endosymbionts (Fig. 4). This result also demonstrates that the variation of endosymbionts changed the culture duration of *P. bursaria* dependently, as depicted in Fig. 3. As presented in Fig. 5A, the cell size and chlorophyll contents of exosymbiotic algae are correlated strongly with the algal cell cycle^46^. For this study, the distribution of endosymbionts in Fig. 4 was categorized provisionally into three populations: a cell population of both small size and high chlorophyll contents (designated as Region I), a population of both large size and high chlorophyll contents (Region II) and a population of low chlorophyll contents (Region III) based on both algal size and chlorophyll contents (Fig. 5B). Here, Regions I, II, and III were assumed respectively as populations of a growing alga, populations including autospores, and populations of a unicellular cell immediately after division. Furthermore, the ratio of algae in each region was estimated and compared to each culture term (Fig. 5C). The ratio of algae at regions I and II increased gradually with incubation time, although those at region III decreased conversely.

### Comparison of population dynamics of endosymbionts in *P. bursaria* with their host paramecia

**Figure 6:**
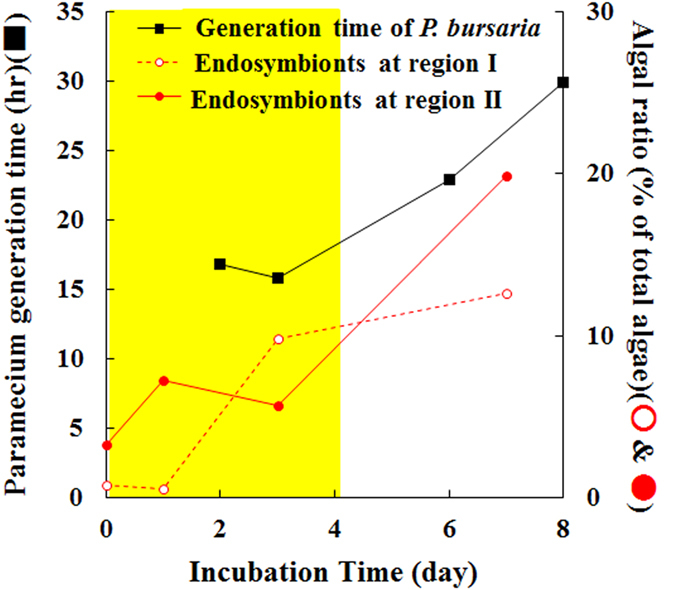
Synchronization of culture time-dependent behaviors between endosymbiotic algae and their host paramecia. *Paramecium* generation time from Fig. 2A and the time-dependent population changes of endosymbionts in *P. bursaria* from Fig. 5C were compared. Yellow and white areas respectively represent the duration for the logarithmic phase and for the early stationary phase of *P. bursaria*.

In general, the algal ratio of each cell cycle phase at some instant is related closely to the actual duration of the cell cycle phase. As described above, the generation time of *P. bursaria* was delayed culture-condition-dependently (Fig. 2A). This study compared the time-dependent population changes of endosymbionts in *P. b*ursaria (Fig. 5C) with the *Paramecium* generation time (Fig. 2A). When *P. bursaria* host cells were at the logarithmic phase, FCM was able to detect little algae at regions I and II before division (Fig. 6). In contrast, more algae at regions I and II were estimated (Fig. 6) when host cells were at the early stationary phase. That result leads ultimately to the delayed generation time of endosymbionts. Consequently, results confirmed the synchronization of culture-time-dependent behaviors between endosymbiotic algae and their host paramecia.

## Discussion

Recently, FCM has offered powerful and effective procedures to elucidate numerous biological, medical, and bioengineering challenges. Most flow cytometers use hydrodynamic focusing with sheath flow to analyze particles such as cells one-by-one. However, FCM has not been used to analyze symbiosis models directly because representatively complex symbioses models such as *P. bursaria, H. viridissima*, and coral are sufficiently larger than suitable cells for FCM measurements. A recent study detected intact *P. bursaria* cells using capillary FCM, not hydrodynamic focusing with sheath flow fluid^44^. In addition to the detection of *P. bursaria* host cells, this study was intended to examine life cycle behavior between the host and the endosymbionts in *P. bursaria* using capillary FCM. In fact, the capillary FCM in this study was able to detect subtle changes at the microscopic level and to discriminate *P. bursaria* cells at the interphase from those at the division phases (Fig. 2G). The FCM system was also able to discriminate endosymbionts from the host *P. bursaria* using FSS and red fluorescence intensity, as described in an earlier study^44^. Technical advantages of microcapillary FCM over other conventional techniques are the implementation of simultaneous detection and evaluation of both endosymbionts and their host *P. bursaria*. Moreover, no need exists for any special size nozzle. Many time-saving and sensitive procedures are therefore available to conduct symbiosis studies.

A single-cell analysis of *P. bursaria* using microscopy revealed that the number of endosymbionts almost doubles when the host cell of *P. bursaria* is at the division phase^15^,^45^. This result demonstrates that the host cell of *P. bursaria* strictly controls the number of endosymbionts in one way or another. Endosymbionts must arrange their cell cycle schedule accordingly if the culture-condition-dependent change delays the generation time of *P. bursa*ria (Fig. 2A). However, it remains poorly understood whether the endosymbionts act in concert with the culture-condition-dependent behaviors of *P. bursaria*. From data of both *Paramecium* numbers using microscopy and those of endosymbionts using FCM, this study investigated the life cycle behaviors of endosymbionts and the host *Paramecium*. To establish a connection between the host cell cycle (Fig. 2) and multivariate data of endosymbionts obtained using FCM comprehensively, the PCA method was applied (Fig. 3). That method derived two important factors related to the connection between the host and the endosymbionts cell cycles from data: the endosymbiont size and chlorophyll fluorescence intensity. Graphs of size versus chlorophyll fluorescence of endosymbionts detected that the ratio of algae after division decreased gradually over time, instead of those before division increasing (Fig. 4). Results show that endosymbiotic algae in regions I and II divided immediately when the host *Paramecium* was at the logarithmic phase (Figs 4, 5 and 6). Algae in regions I and II divided instantly. A smooth transition of endosymbionts occurred from algae at the growing and division phases (regions I and II) to algae at region III. In contrast, endosymbiotic algae in regions I and II stagnated when the host *Paramecium* was at the stationary phase (Figs 4, 5 and 6). It also showed that algae at regions I and II divided at a crawl and that slow transition of endosymbionts occurred from algae at the division phase to algae at region III. The relation between the host *Paramecium* and the endosymbiotic algae, which behave like chloroplasts, is analogous to that between a photosynthetic eukaryotic cell and their chloroplast(s). The synchronous division between chloroplasts and algae in most algal species is achieved by expression control of the nucleus-encoded chloroplast division genes and proteins^9^,^10^. In practice, most chloroplast genes derived from ancestral cyanobacterium have been lost or relocated to the host nucleus through EGT events^6^,^7^,^8^,^9^,^10^. Therefore, organelles such as chloroplasts no longer survive outside of the eukaryotic cell. In contrast to organelles, exosymbiotic algae isolated from *P. bursaria* can proliferate independently from the host ciliate^32^. That capability demonstrates that algae isolated from *P. bursaria*, unlike organelles such as chloroplasts, can self-manage their cell cycle pace. However, these results support the inference that endosymbionts find it necessary to hasten or slow their cell cycle pace with their host cell cycle and that a synchronous life cycle between the endosymbionts and the host *Paramecium* is guaranteed in the *P. bursaria* symbiosis system.

**Figure 7:**
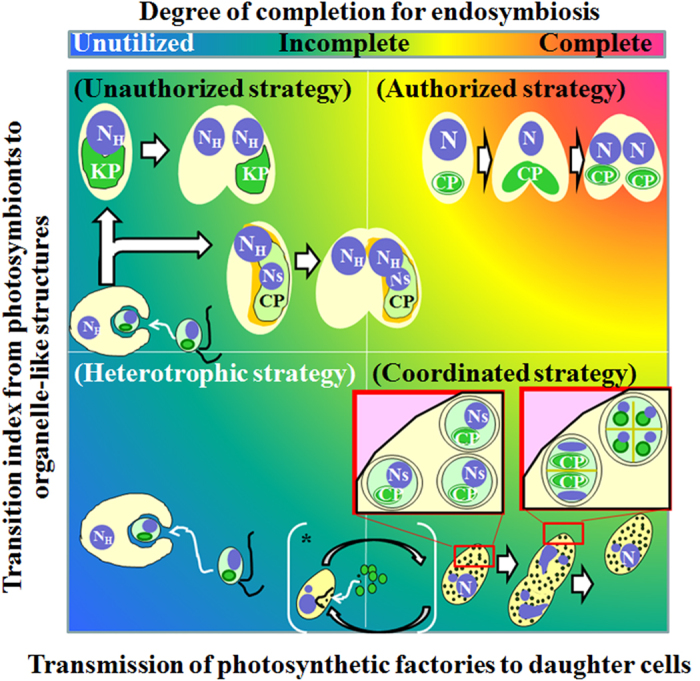
Evaluation of endosymbiosis status for the transition from photosymbionts to organelle-like structures vs. transmission of photosynthetic factories as plastids or intact algae to daughter cells. The graph was quartered: Organisms use organelle-like structures such as plastids in upper areas, whereas they use eukaryotic algae as photosynthetic factories but not plastids in lower areas. Organisms can transmit their photosynthetic factories to daughter cells as shown right areas, although not in the left areas. Consequently, a lower left area presents heterotrophs only, whereas the upper right shows those using an authorized strategy like photosynthetic organisms from primary, secondary, and tertiary endosymbioses. Areas other than secondary and tertiary endosymbioses are multiple endosymbioses. The abbreviated words N_H_, N_S_, KP and CP in Fig. 7 respectively indicate each host nucleus, each nucleus of their symbionts, each kleptoplast derived from their algal prey and each chloroplast.

This study compared the synchronous division cycle strategies of endosymbionts and the host in *P. bursaria* with several strategies in other symbiosis systems (Fig. 7). To realize synchronous division between organelles and their host cells, sophisticated strategies including EGT and LGT have been performed. Particularly, LGT is an important factor in establishing a stable endosymbiosis with other eukaryotes because the new host cell can take over the function from EGT-containing organelle division genes by LGT. Actually, LGT and synchronization of division cycles in several ongoing endosymbioses (upper left and lower right in Fig. 7) are apparently less successful than well-established and integrated symbioses in secondary and tertiary endosymbioses (upper right in Fig. 7). Several photosynthetic animals including sacoglossan sea slugs have evolved to acquire only plastids (kleptoplasts) from algal prey^4^. However, they seem to neglect or fail to obtain endosymbiotic related genes as LGT from the algal prey. In fact, the plastids are not transmitted vertically and do not undergo division in the sea slugs^4^. Rauch *et al*.^31^ concluded from RNA-seq analysis that there is no evidence that the evolution of kleptoplastry in sea slugs involves LGT events. The plastid from endosymbionts seems also not to undergo division in the marine ciliate *M. rubrum*^25^,^26^,^27^,^28^, which sustains not only plastids but also a transcriptionally active nucleus from algal prey. Therefore, it is considered that *M. rubrum* may need to feed periodically to replace ageing plastids or plastids diluted out by host cell division^25^. New individuals of these photosynthetic animals and ciliates must necessarily acquire new plastids from algal prey to obtain photosynthetic benefits. The endosymbiosis in these photosynthetic organisms is apparently an unauthorized strategy without LGT events or with incomplete LGT. The ongoing plastid-acquisition types of endosymbiosis are defective in their ability to maintain permanent endosymbiosis compared to secondary and tertiary endosymbioses (upper left in Fig. 7). In contrast to the transient endosymbioses explained above, the endosymbiosis in *P. bursaria* seems to apply a coordinated strategy by which endosymbionts fine-tune their cell cycle pace with their host *Paramecium* (Fig. 6 and lower right in Fig. 7). In practice, some endosymbiotic algae can at least undergo cell division and are transmitted to daughter cells. Furthermore, *P. bursaria* has the capability of re-establishing the endosymbiosis between the host and their endosymbionts for emergency, even if the host cell loses the endosymbiotic algae^32^,^37^,^38^ (*, lower middle in Fig. 7). All intact alga-embracement types, however, are not always coordinated and are not superior to the plastid-acquisition types of endosymbiosis. A katablepharid flagellate, *Hatena arenicola*, has an endosymbiotic alga, which is slightly atrophied rather than intact^8^. When the flagellate host cell is at the division phase, the endosymbiont does not divide synchronously with the host. After host cell division, the flagellate harboring the endosymbiont and the other with no endosymbiont are produced^8^ (upper middle in Fig. 7). It is particularly interesting that the daughter cell with no alga can uptake the partner alga and can re-establish endosymbiosis between the host and the endosymbiont^8^ just as algae-free *P. bursaria* can, although the appearance of algae-free *P. bursaria* is practically from artificial conditions^34^,^35^,^36^,^37^. The basal metazoan *H. viridis* also has endosymbiotic algae^12^,^17^,^18^. The symbiotic partners are necessary for the hydra sexual reproduction, particularly for the production of female gonads^18^, although the presence or absence of endosymbiotic algae is unaffected in *P. bursaria*^47^. Moreover, *P. bursaria* host cells are ready to cooperate with photosymbiotic partners in tolerance to reactive oxygen species, which might be generated from energetic metabolism containing photosynthesis, even if algae-free *Paramecium* is^43^,^48^. Considering several endosymbioses, the endosymbiosis in *P. bursaria* is coordinated fortuitously with endosymbionts, not so much with control of endosymbiotic algae by the host, as with that of organelles such as chloroplasts, for photosynthetic function and transmission of endosymbionts after cell division.

To elucidate symbiosis in *P. bursaria*, the effects of some chemicals such as protein synthesis inhibitors^34^,^35^,^36^,^49^ and cytoskeletal depolymerization inhibitors^15^ on *P. bursaria* were examined microscopically. Studies based on microscopic observation have generally involved great amounts of time for obtaining data. Moreover, they have not been done with high-throughput experiments. FCM can save time better than microscopic observations can. When Gerashchenko *et al*.^42^ first introduced FCM to study the endosymbiosis of algae in *P. bursaria*, almost all commercially available flow cytometers applied hydrodynamic focusing with sheath fluid in the biomedical markets of that time. Therefore, instead of analyzing *P. bursaria* host cells directly, exosymbiotic algae isolated from their host paramecia were the main target of FCM measurements^42^,^46^. However, their algae are far from airtight experiment materials to elucidate the mechanisms of endosymbiosis because algae have not been controlled stringently by the host. Newly and commercially supplied capillary FCM was able to detect intact *P. bursaria*^44^. Although the hydrodynamic focusing system has been superior to the microcapillary system in terms of resolution ability to detect small cells, this system with microcapillary technology holds enormous potential for application to studies of symbioses such as those of *P. bursaria*. In addition to detecting endogenous fluorescence of their algae, the combination of fluorescence labeling antibodies for cell cycle dependent biomolecules and flow cytometry might provide valuable insights to support symbiosis studies. Taken together, this system with microcapillary technology can offer a powerful tool for studies of symbiosis and evolution of eukaryotic cells.

## Methods

### Strain and culture condition of *P. bursaria*

After collecting *P. bursaria* from a river Asida-kawa (Hiroshima prefecture, Japan), paramecia were cultured in a lettuce infusion containing *Klebsiella pneumonia* bacteria one-by-one into a culture dish. A cloned *P. bursaria* syngen I (AS-2, mating type IV) of paramecia was used for this study. The cloned paramecia were cultured in lettuce infusion containing *K. pneumonia* as food under an LD cycle (12 h light/12 h dark) at *ca.* 1100 lux natural white fluorescence light and 23 ± 2 °C.

### Culture condition of *Chlorella*-like exosymbiotic algae isolated from *P. bursaria*

*Chlorella*-like exosymbiotic algae (SA-1 strain) isolated from *P. bursaria*^32^ were used. The cloned strain of exosymbiotic algae on an agar plate containing CA medium^32^ were cultured under a constant light condition at 3500 lux of natural white fluorescent light and 25 ± 1 °C.

### Growth curve of *P. bursaria*

Paramecia at the logarithmic to stationary phase were collected and counted using a stereomicroscope. Paramecia at an initial density of 20 cells/ml were cultured in fresh lettuce medium containing *K. pneumonia* under an LD cycle (12 h light/12 h dark) at *ca.* 1100 lux of natural white fluorescence light and 24 °C. The paramecia (average ± standard deviation) were counted over time using stereomicroscopy. The generation time (g) (hr) was calculated from data obtained as given in equations (1)–(3).


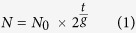



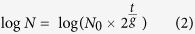



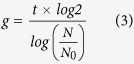


Here, *N*_0_ and *N* respectively denote the initial density of paramecia (cells/ml) before incubation and the numbers of paramecia (cells/ml) after incubation. Therein, *t* denotes the incubation time (hr). Therefore, 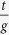 represents the frequency of cell division.

### Preparation of specimens for light microscopy

Paramecia were collected and fixed with 5% (v/v) formalin. Microscopic images were made of collected paramecia fixed with 5% formalin.

### Evaluation for *P. bursaria* size using microcapillary FCM

To estimate the *Paramecium* size, a microcapillary flow cytometer (Muse^TM^ Cell Analyzer; Merck Millipore), but not one based on hydrodynamic focusing with sheath flow, was used as described in an earlier report^44^. *P. bursaria* fixed with 5% (v/v) formalin was analyzed at flow rates of 0.59 μl/s (sample load time <2 min/single measurement). Cells that went through the rectangular capillary with 100-μm round bore were analyzed using this instrument equipped with a green laser operating at 532 nm for several optical properties. FSS were collected to ascertain cell size. The fluorescence of chlorophyll was detected in the red fluorescence channel through a 680/30 nm band pass filter simultaneously. To obtain sufficient data including intact *P. bursaria*, FCM measurements were repeated several times (5–20 times). The paramecium amounts in the logarithmic phase did not yet reach sufficient numbers. Therefore, the shorter the culture duration was, the more repeated measurements for intact *P. bursaria* cells were needed.

Individual signals in each FCS3.0 file were extracted using software (FCSExtract utility ver. 1.02; Stowers Institute for Medical Research). They were subsequently re-analyzed using standard spreadsheet software (Access and Excel; Microsoft Corp.). When *P. bursaria* cells with endosymbiotic algae were analyzed using FCM, two distinct populations were detected (designated as R1 for intact *P. bursaria* cells and R2 for their algae released from several broken *P. bursaria* cells, respectively) as described in the previous report^44^. The size variation in *P. bursaria* was expressed as a distribution histogram of relative size from FSS.

### Evaluation of size and chlorophyll contents of endosymbiotic algae in *P. bursaria* using microcapillary FCM

To estimate the optical properties of endosymbiotic algae, the microcapillary FCM was used as described above. The earlier report described that the passage rate of intact *P. bursaria* through the capillary was 79 ± 9.9% 44. A *P. bursaria* cell at interphase has *ca*. 300–500 endosymbiotic algae in cytoplasm^15^,^45^. Consequently, the capillary FCM was able to analyze a statistically sufficient number of endosymbiotic algae released from several broken *P. bursaria* during FCM measurements. Whereas the size and chlorophyll contents of *P. bursaria* host cells were evaluated from the R1 population as described in the previous report^44^, those for endosymbiotic algae were evaluated simultaneously from the R2 population.

### Correlative evaluation using PCA of multivariate analysis

This study was undertaken to evaluate the correlativity between host and algal life cycles over time. To evaluate the correlativity between multiple properties including incubation time of *P. bursaria*, and the size and chlorophyll contents of endosymbiotic algae, PCA of multivariate analysis was used for this study. As a dimensional reduction technique, PCA reduces multiple-dimensional information to arbitrary one-dimensional information, which is a dataset from a new axis produced by PCA. For this study, the dataset produced from FCM data was subjected to PCA analyses using software for multivariate analysis (Institute of Statistical Analyses, Inc.). Based on results of the correlation matrix analysis for the data obtained, the author calculated the contribution rates of respective components, the factor loading of each parameter, and the score plot of each component were calculated. Here, each factor loading indicates correlation factors between each parameter and each component (PC1-3).

### Interim evaluation for cell cycle of endosymbiotic algae in *P. bursaria*

Previous studies determined the algal cell cycle from the size and chlorophyll contents of exosymbiotic algae isolated from *P. bursaria*^46^. After detection of signals for endosymbiotic algae released from *P. bursaria*, an interim evaluation for the cell cycle of endosymbiotic algae was done using signals of the size and chlorophyll contents. Endosymbionts were categorized into three populations: cell population of both small size and high chlorophyll contents (<75 on the forward scatter signal and ≥15 on the red fluorescence intensity) (region I); the population having both large size and high chlorophyll contents (≥75 on the forward scatter signal and ≥15 on the red fluorescence intensity) (region II); and the population with low chlorophyll contents (<15 on the red fluorescence intensity)(region III). Then, the respective time-dependent change ratios of algal numbers for all regions were estimated at different incubation times of *P. bursaria*.

## Additional Information

**How to cite this article**: Takahashi, T. Simultaneous Evaluation of Life Cycle Dynamics between a Host *Paramecium* and the Endosymbionts of *Paramecium bursaria* Using Capillary Flow Cytometry. *Sci. Rep.* **6**, 31638; doi: 10.1038/srep31638 (2016).

## Figures and Tables

**Figure 1:**
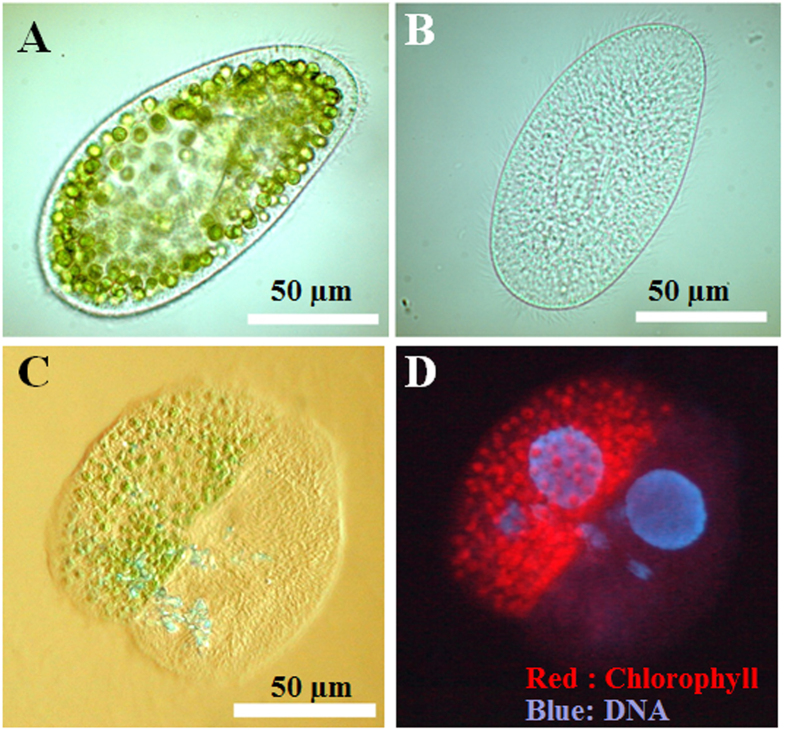
Examples of *P. bursaria* in a natural state and some experimental states. (**A**) *P. bursaria* in a natural state. (**B**) Algae-free *Paramecium* produced by treatment with paraquat herbicide, as described previously^34^. (**C**) Sexual reproduction and conjugation of natural *P. bursaria* with algae-free *Paramecium* produced by treatment with acrylamide, as described previously^35^. (**D**) A fluorescence image of panel C is shown. Red and blue fluorescence derive, respectively, from endogenous chlorophyll of endosymbiotic algae and from DAPI-staining *Paramecium* nuclei.
